# Combined Effect of Drying Temperature and Varied Gelatin Concentration on Physicochemical and Antioxidant Properties of Ginger Oil Incorporated Chitosan Based Edible Films

**DOI:** 10.3390/foods12020364

**Published:** 2023-01-12

**Authors:** Saurabh Bhatia, Ahmed Al-Harrasi, Sana Ullah, Mohammed Said Al-Azri, Alaa El-Din Ahmed Bekhit, Layal Karam, Mohammed Albratty, Mohammed F. Aldawsari, Md. Khalid Anwer

**Affiliations:** 1 Natural & Medical Sciences Research Center, University of Nizwa, 616 Birkat Al Mauz, Nizwa P.O. Box 33, Oman; 2 School of Health Science, University of Petroleum and Energy Studies, Prem Nagar, Dehradun 248007, India; 3 Food Science Department, University of Otago, Dunedin 9056, New Zealand; 4 Human Nutrition Department, College of Health Sciences, QU Health, Qatar University, Doha P.O. Box 2713, Qatar; 5 Department of Pharmaceutical Chemistry and Pharmacognosy, College of Pharmacy, Jazan University, P.O. Box 114, Jazan 45142, Saudi Arabia; 6 Department of Pharmaceutics, College of Pharmacy, Prince Sattam Bin Abdul Aziz University, P.O. Box 173, Al-Kharj 11942, Saudi Arabia

**Keywords:** edible films, polymer, chitosan, essential oil, gelatin, drying temperature, food packaging

## Abstract

In the present work, ginger essential oil (GEO) loaded chitosan (CS) based films incorporated with varying concentrations of gelatin (GE) were fabricated and dried at different conditions (25 °C and 45 °C). The physio-chemical, mechanical and antioxidant potential of the films were determined. Films dried at 45 °C showed better physical attributes and less thickness, swelling degree (SD), moisture content, water vapor permeability (WVP), more transparency, and better mechanical characteristics. Fourier transform infrared spectroscopy (FTIR) revealed the chemical composition and interaction between the functional groups of the film components. X-ray diffraction (XRD), thermogravimetric analysis (TGA), and scanning electron microscopy (SEM) findings revealed that samples dried at 45 °C had more crystalline structure, were thermally stable, and smoother. Antioxidant results showed that films dried at low temperature showed comparatively more (*p* < 0.0001) antioxidant activity. Additionally, an increase in gelatin concentration improved the tensile strength and swelling factor (*p* < 0.05), however, had no significant impact on other parameters. The overall results suggested better characteristics of GEO-loaded CS-GE based edible films when dried at 45 °C.

## 1. Introduction

Edible films (EFs) offer a wide range of benefits to packed food material in terms of preserving its quality and safety profile. These protective materials can effectively reduce the waste and food degradation caused by microbial contamination, oxidation, moisture losses and undesirable chemical reactions [1]. Additionally, these materials can be loaded with a range of food additives with potent antimicrobial and antioxidant materials. These edible, eco-friendly and nontoxic materials offer considerable benefits in terms of offering favorable optical, barrier and mechanical properties. However, biopolymer based films always present certain demerits in terms of barrier, mechanical, and physicochemical properties [2]. To overcome the limitations of biopolymer based edible films made from a single biopolymer, several composite materials have been studied by using them in combination with polysaccharides (sodium alginate, cellulose, starch, pectin, chitosan), proteins (gelatin, gluten, soy protein, casein, whey protein), and lipids (waxes) [1,3]. Recent studies showed the increasing interest in the fabrication of composite material by using a secondary polymer with the aim of overcoming the challenges faced by the primary polymer [4]. Chitosan is a cationic polysaccharide that possesses excellent film forming, mechanical, and barrier properties (it has selective permeability to gasses such as O_2_ and CO_2_). Nevertheless, chitosan based films showed permeability to water vapor, which restricts its utilization in the food industry. Previous research showed that chitosan in combination with different ratios of polysaccharides and proteins offer films with improved properties [5]. Gelatin based films have been reported for their exceptional emulsifying, gel forming, UV protection, and film forming properties and this polymer has been utilized to form a uniform film forming solution with chitosan. Chitosan-gelatin composite blends have demonstrated good mechanical, thermal, surface hydrophobic, optical and barrier properties [6,7,8,9,10].

Gelatin based films have been frequently reported due to its best film forming properties. Gelatin has the ability to form uniform solutions with chitosan and other composites and have demonstrated good mechanical, thermal, hydrophobic, optical and barrier properties [6,7]. Earlier studies suggested that an increase in the concentration of gelatin up to a certain level can improve the physical and chemical properties of the film [9,10]. Loading these films with food additives and functional ingredients was reported to be a reliable approach to improve the physical, chemical, and biological properties of the films [11]. A recent trend in loading composite films with essential oils not only improves the antioxidant, antimicrobial, and other properties of the films but also imparts a unique flavor and aroma to a wide range of food products [12]. Ginger essential oil (GEO) is known for its broad range of benefits including antioxidant, antimicrobial, and other biological properties. According to the Food and Drug Administration (FDA), this oil is considered “generally recognized as safe”, and hence, it is suitable for food applications [13]. In the present study, chitosan-gelatin (CS-GE) films were loaded with GEO to improve their overall properties.

Conditions used during the fabrication of EFs impact their physical and chemical properties considerably [14]. For example, the film drying temperature impacts the polymeric interactions, overall crystallinity, and reorganization of the films [15]. Subsequently, composite films dried at different drying temperatures can have different barrier, mechanical, and antioxidant properties [16]. There are few studies that describe the effects of different concentrations of gelatin on the physical and chemical properties of GEO-loaded CS based EFs [17].

Therefore, the present study aimed to develop GEO incorporated CS-GE films and to study their behavior at different GE concentrations and drying temperatures.

## 2. Materials and Methods

### 2.1. Materials

Gelatin-Type B (Porcine) and CS (extra pure, 150–500 m. Pas, with 90% degree of acetylation) were procured from SRL Pvt Ltd., Mumbai, India. *Zingiber officinale* oil (100 mL) was obtained from Nature Natural, Ghaziabad, India. Acetic acid, 2,2′-diphenyl-1-picrylhydrazyl (DPPH), Folin-Ciocalteu phenol reagent, ferric chloride, Trolox (6-Hydroxy-2,5,7,8-tetramethylchromane-2-carboxylic acid), butylated hydroxyl anisole and potassium persulfate were procured from Sigma-Aldrich, London, UK.

### 2.2. Preparation of CS-GE-GEO Films

To analyze the effect of various concentrations of gelatin (1–1.5%) over the behavior of GEO loaded CS films at different drying temperature conditions (25 °C and 45 °C), films including blank (CS, CS-GE composite) and GEO loaded (CS, CS-GE composite) were fabricated using the casting method. Initially 500 mL solution of CS 1% (*w*/*v*) (with 90% degree of deacetylation) was made by using 1% of acetic acid (*v*/*v*) followed by the addition of glycerol (10%). The resultant CS solution was divided into ten parts (into glass beakers) and coded as FL1–FL10. FL1 and FL6 (blank CS solution) were poured into plastic petri plates and then dried at 25 °C (24 h) and 45 °C (12 h). GEO (1.5%, *v*/*v*) was added to the blank CS solution in FL2 and FL7 and later dried at different temperatures (25 °C and 45 °C). Subsequently, 1% (*w*/*v*) of gelatin was added to FL3 and FL8 and stirred (6 h) until complete dissolution of both polymers via a magnetic stirrer. This was followed by drying at 25 °C and 45 °C. GE (1% *w*/*v*) and GEO (1.5%, *v*/*v*) were incorporated into a film forming solution of CS (1% *w*/*v*) and dried at 25 °C and 45 °C to form FL4 and FL9. Similarly, GEO (1.5% *v*/*v*) and GE (1.5% *w*/*v*) were added into a film forming solution of CS (1% *w*/*v*) and dried at 25 °C and 45 °C to form FL5 and FL10. After drying, the edges of the films were treated with 90% ethanol for easing the peeling off of the film from the surface of the petri dish. Dried films were stored at ambient conditions (at 45 ± 2% RH and 25 ± 2 °C) prior to the characterization. After preparation, all films were screened and assessed by visual characterization. Table 1 shows complete information regarding the films’ composition.

### 2.3. Thermogravimetric (TGA) Analysis

TGA analysis of the prepared samples was assessed using a thermal analyzer (SDTQ600, TA instruments, New Castle, DE, USA). Composite films were scanned from room temperature to 600 °C at a heating rate of 10 °C/min under constant purging with nitrogen gas. Zinc was used as the metallic standard.

### 2.4. X-ray Diffraction (XRD)

XRD curves of the prepared samples was reported by using a Bruker D8, Discover instrument, Karlsruhe, Germany. Each sample was examined at diffraction angle 2θ ranging from 5–50° at 40 kV.

### 2.5. Microscope Observations

The morphology (surface and cross section) of samples (FL1–FL10) were analyzed using a scanning electron microscope (SEM) (JSM6510LA, Analytical SEM, Jeol, Akishima, Tokyo, Japan) under high vacuum mode at 20 kV. All of the samples were mounted over aluminum stubs with adhesive tapes and sputter-coated with gold.

### 2.6. FTIR Spectra Analysis

Chemical changes among the different samples were analyzed using a Fourier transform infrared Bruker Tensor 37 spectrometer (Ettlingen, Germany). FTIR measurements were obtained at 25 ± 1 °C, with a resolution of 4 cm^−1^ and 32 scans and in the wavenumber range of 400 cm^−1^ to 4000 cm^−1^.

### 2.7. Thickness

The thickness of the EFs greatly influence the optical, barrier, and mechanical properties. In the present work, the films’ thickness (in mm) was evaluated by a manually operated micrometer (Mitutoyo digital micrometer 2046F, Mitutoyo, Kawasaki, Japan) having a precision of ± 0.001 mm at 5 randomly selected points prior to mechanical testing.

### 2.8. Mechanical Properties

Mechanical testing of the fabricated films was determined by tensile strength (TS), Young modulus (Ym) and elongation at break (EAB) using the Texture Analyzer (Stable Micro System, Godalming, UK). For assessing the mechanical properties of the samples, the ASTM (1995) standard operating procedure was used [18].

To assess the mechanical properties, the film sections were sliced into strips with dimensions of 10 mm × 40 mm. Later on, these rectangular strips were conditioned at 50% relative humidity for 72 h. When the films were affixed into the grips (at 0.75 mm/s until break) and showed the extension at this point, the force and distance values were determined. The TS was assessed by maximum force at break by the original cross-sectional area (m × m) of the EFs, whereas the elongation at break (EAB%) was estimated by dividing the elongation at the instant of break by the preliminary gauge measurement and multiplying this by 100. All the measurements were performed in triplicate and the mean values were selected.

### 2.9. Water Vapor Permeability (WVP)

For WVP evaluation, the ASTM (2003) standard procedure was followed. For this purpose, EFs without any perforation were selected and cut into a round shape with a diameter of 20 mm. These films were used to seal glass cups containing silica gels, which were stored at zero percent relative humidity. In addition, after sealing, these cups were kept in desiccators (at 25 °C) comprising distilled water (RH 100%) and weighed at 60 min intervals over 10 h. All the measurements were performed in triplicate; the following calculation was used to evaluate the WVP (ASTM, 2003) [19]:

WVP = w/t × t/(ΔD × FA)(1)

where w/t is the weight loss in moisture content/unit of time, (gs-1) is determined by linear regression (R2 > 0.99) from the water absorbed by the system when a stable state was achieved. FA is the area of the film exposed against moisture transfer (1.539 × 10^−4^ m^2^), t, is the mean film thickness, and ΔD is the difference in water vapor pressure between the two sides of the film at 25 °C (kPa).

### 2.10. Water Solubility (WS)

For the water solubility (WS) analysis, the Singh et al. 2015 procedure was followed with a little modification [20]. The WS of the samples was calculated as the amount of dry matter solubilized after 24 h of dipping in water. To determine the solubility of the films in the water, 250 mg of film was transferred into 25 mL of water (at 25 ± 1 °C) for 24 h and the solution was stirred at 300 rpm (25 ± 1 °C). Insoluble film fragments obtained from this solution were dried using a hot air oven (at 105 ±  1 °C) till constant weight was attained. Furthermore, the swelling degree was determined by using the Nazan T et al. 2004 method [21].

Solubility (S) was determined (in %) by the following equation.

S (%) = [(Wi − Wf)/Wi] × 100(2)

The Wi and Wf represent the weight of the initial dry film and the weight of the undissolved films.

### 2.11. Moisture Content

Moisture content was evaluated by assessing the variation in weight after drying at 105 °C for 5 min using a KERN moisture analyzer.

### 2.12. Swelling Degree

The swelling factor was calculated as the amount of water (mL) absorbed by 1 g of film sample. The test was performed in triplicate and the mean value was used for evaluation. The Basiak E et al. 2015 method was followed for the assessment of the swelling factor [22]. The films (2 × 2 cm) were initially weighed (W1) and then immersed in a flask with distilled water at room temperature for 2 min. Later on, the films were transferred from the flask to the filter paper to remove the excess water. Again, the samples were weighed (W2) and the quantity of adsorbed water was evaluated in percent.

### 2.13. Light Transmission

Transparency of the fabricated samples was evaluated by a UV–visible spectrophotometer (UV160U-Shimadzu, Kyoto, Japan) at 550 nm. The samples were sliced into a rectangular shape with a dimension of 3 × 15 mm and transferred into cuvette. A cuvette without film was considered a blank. Measurement was carried out using the following formula:

Transparency (%) = Film Thickness (mm)/absorbance of the sample (nm)

### 2.14. Total Phenol Content

For the assessment of total phenolic content (TPC), the Folin–Ciocalteu reagent was used [23]. The findings were presented in mg gallic acid equivalent (GAE)/g dry weight. Mean values were obtained from the triplicates.

### 2.15. Antioxidant Assays

Sample preparation and antioxidant assessment were performed according to Gómez-Estaca et al. 2009 with slight modifications [24]. For the sample preparation, a 500 mg of EFs sample was weighed, dissolved in 1% acetic acid, and then transferred to a flask containing methanol (15 mL). The solution obtained was mixed for 1 h using a rotary shaker at 100 RPM. The resultant solution was subjected to centrifugation at 1000 RPM for 10 min at 4 °C. Lastly, to assess the total phenolic content and the antioxidant properties, the supernatant layer was separated from the solution.

#### 2.15.1. DPPH Radical Scavenging Assay

The 2,2-diphenyl-1-picrylhydrazyl (DPPH) free radical scavenging activity of the film samples was determined by following the Ruiz-Navajas et al. 2013 method with a few changes [25].

#### 2.15.2. ABTS Assay

The ability of the EFs to scavenge 2,2-azinobis-(3-ethylbenzothiazoline-6-sulfonic acid (ABTS+)) radicals was determined using the Re R et al. 1999 procedure [26].

### 2.16. Statistical Analysis

A one-way analysis of variance followed by Duncan’s test (mechanical and barrier parameters) and Tukey’s test (antioxidant parameters) were used to find differences within the groups.

## 3. Results and Discussion

### 3.1. Visual Appearance of the EFs

**Figure 1:**
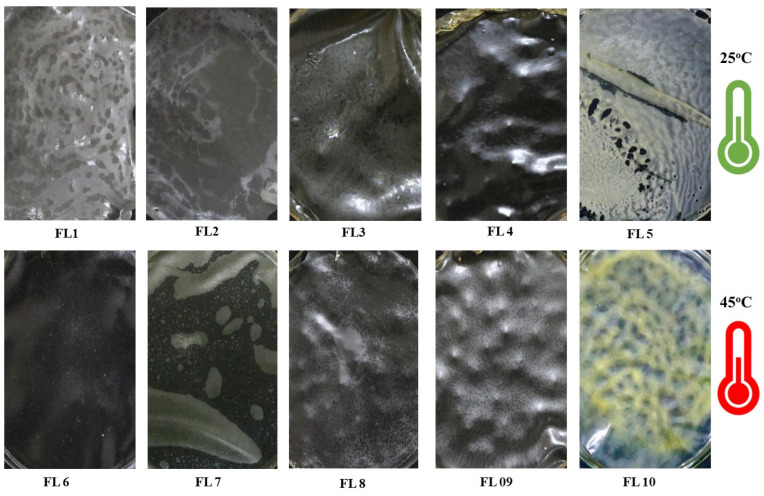
Visual appearances of edible films FL1–FL10.

It is important to assess the visual differences (color, stickiness, rigidity, surface texture, transparency, uniformity) among the films dried at different temperature conditions. This was carried out as the initial screening step of the films. Table 2 and Figure 1 show the visual characterization of the EFs dried at 25 °C and 45 °C. Overall, it was observed that samples dried at higher temperature demonstrated better physical attributes, such as surface roughness, mechanical strength, color, transparency, uniformity, and flexibility, than films dried at lower temperature. In addition, films dried at higher temperature peeled out easily from the petri plates’ surface and showed less shrinkage than films dried at lower temperatures.

### 3.2. Thermogravimetric Analysis

**Figure 2:**
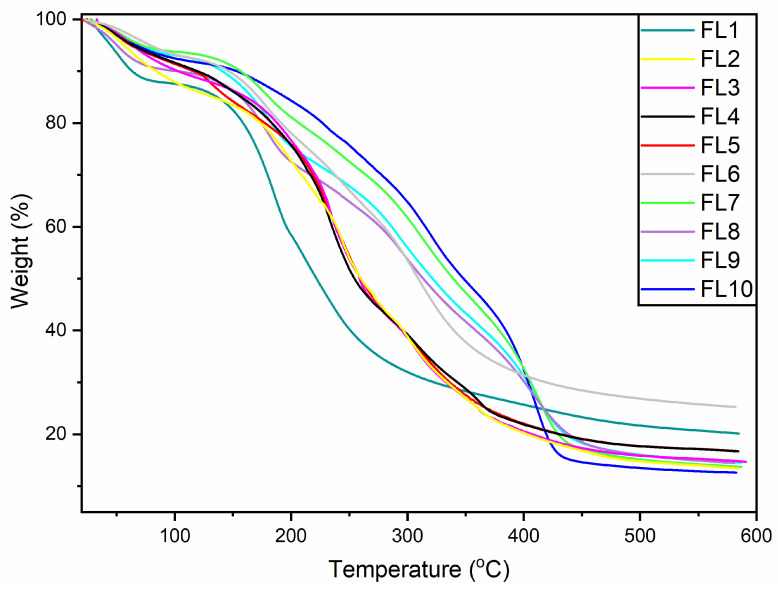
Thermogravimetric analysis (TGA) spectra of blank and composite films (FL1–FL10). FL1–FL5; EFs dried at 25 °C, FL6–FL10; EFs dried at 45 °C.

Thermogravimetric analysis (TGA) was carried out to evaluate the thermal stability and weight degradation pattern of the films. The TGA findings revealed multistage thermal decomposition of the samples (FL1–FL10) similar to previous reports [27]. The blank films, which contained only CS, demonstrated a sharper thermal decomposition pattern than the others, similar to previous studies [15]. During the first stage (26–100 °C), all the edible films (FL1–FL10) behaved similarly in terms of the thermal decomposition pattern (Figure 2). Around 9–10% weight loss was recorded in the edible films at a temperature range of 26–100 °C. Weight loss at this stage could be due to the water molecules present in the films [28].

In the second stage, an apparent weight loss was observed, which started at 125 °C for all the samples, while the final temperature was found to be different for some of the films. Weight variation at this phase could be due to the thermal decomposition of the main components of the films, mainly CS and GE [29]. The thermal decomposition pattern of the samples within the groups was found to be similar (Figure 2). However, both groups (FL1–FL5 and FL6–FL10) showed different thermal decomposition patterns from each other. Among FL6–FL10, the final temperature of the thermal decomposition was around 457 °C, with 72% weight loss. The weight loss pattern of the film FL1, was found to be different (64% weight loss) from the rest of other films at this stage. Overall, the TGA pattern of the blank films (FL1 and FL6) was little different from the others in their respective groups. This could be attributed to the chemical composition of FL6. Moreover, it was revealed that sharp weight loss was observed in the FL1–FL5 group compared to the FL6–FL10 group, which demonstrated a steady weight loss pattern. The higher thermal stability in the FL6–FL10 group could be associated with a strong molecular interaction between the film components at higher temperature [28]. The complete results suggested that samples dried at 45 °C showed more thermal stability compared to films dried at lower temperature.

### 3.3. X-ray Diffraction (XRD)

**Figure 3:**
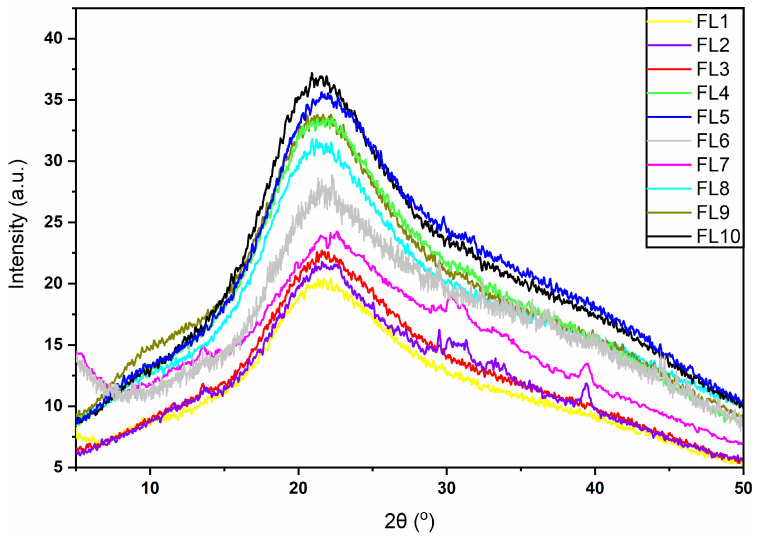
XRD Pattern of EFs (FL1–FL10). FL1–FL5; EFs dried at 25 °C, FL6–FL10; EFs dried at 45 °C.

XRD analysis was performed to study the compatibility between the components (CS, GEO, GE) of the films as well as to assess the crystallinity and amorphousness of the films (FL1–FL10) fabricated at various conditions [30]. The XRD findings of the synthesized EFs revealed characteristics peaks with varied intensities, showing the effect of different drying conditions. The diffraction patterns of all the EFs containing different components were found to be similar at 21° of 2θ (in between the 15.5–28.6° positions). Figure 3 shows small characteristic peaks at the 29° and 39° position of 2θ in a few samples (FL2 and FL7), which might be associated with the chemical interaction between CS and GEO. However, the overall crystallinity of the films dried at low and high temperatures were decreased with the addition of GEO.

The XRD results suggested a better chemical interaction between the different chemical components in the composite films. Overall, the XRD peaks observed in all samples were broader, which reveals that these samples contained fewer or partial crystalline structures. Previous studies have also reported a similar XRD pattern when CS and GE based composite films were synthesized [31,32]. The XRD findings revealed that the overall crystallinity of the films increased as drying temperature increased from 25 °C to 45 °C (Figure 3), which could be due to the increase in ordered arrangement of polymeric molecules at higher drying temperature conditions, or the evaporation of GEO at a higher drying temperature [33]. From the current findings, we observed that the difference in drying temperature has little impact on the XRD pattern (peak intensities) of composite films (FL4, FL5, FL9 and FL10), suggesting that there were no changes in the inter-planar spacing of associated crystallites. Thus, this signifies the chemical stability as well as the compatibility between the components of these films. On the other hand, the blank films and GE loaded films showed changes in peak intensities due to the difference in drying temperature. In addition, increasing the concentration of GE from 1 to 1.5% in composite films had no significant difference in the XRD patterns.

### 3.4. Scanning Electron Microscopy

Scanning electron microscopy (SEM) of the blank films (FL1 and FL6), GEO loaded CS films (FL2 and FL7), CS-GE composite films (FL3 and FL8), and GEO loaded CS-GE films (FL4, FL5, FL9 and FL10) containing different concentrations of GE was carried out to analyze the microstructure of the films.

**Figure 4:**
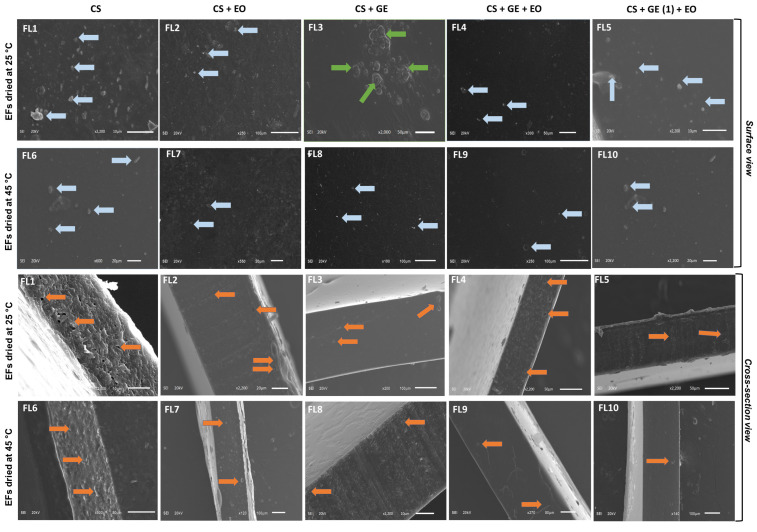
Scanning Electron Microscopy (SEM) analysis of the edible films. FL1–FL5; EFs dried at 25 °C, FL6–FL10; EFs dried at 45 °C. The blue color represents the presence of particles on the films’ surface area; the green color represents ridges; and the orange color represents the presence of pores in the film.

Figure 4 shows the detailed SEM analysis of the films at various magnifications. The addition of GE at different concentrations (1–1.5%) and the effect of different drying temperatures were evaluated. Surface and cross-sectional examination revealed that films (both blank and composite films) dried at a higher drying temperature revealed a favorable surface morphology.

Blank films dried at the low temperature were found to be rough when compared to the blank films dried at the high temperature. On the other hand, the GEO added CS films (FL2 and FL7) revealed a comparatively smoother surface than the CS blank films. This means that the addition of GEO has a positive impact on the surface morphology of the CS based films. CS-GE based films (FL3 and FL8) also revealed good surface morphology at the high drying temperatures. In the FL8 samples, few particles were observed on the surface with fewer pores at the cross-section compared to the counterpart FL3 samples, which were dried at the low temperature.

In addition, the composite films (FL4, FL5, FL9 and FL10) containing CS-GEO, and GE (with different concentrations) revealed better surface morphology (smoother surface with fewer appearances of tiny particles) at a higher drying temperature than films dried at a lower temperature. Moreover, SEM analysis also indicated that the difference in GE concentration had little impact on the surface morphology of films. Similarly, it was observed that increasing GE concentration increased the compactness of the films. FL5 and FL10 were found to be more compact than the other films, which contained a relatively smaller quantity of GE. The SEM findings revealed that films fabricated at higher drying temperature (45 °C) showed better surface morphology than those dried at lower temperature (25 °C). This response is possible due to the increase in the interaction among the different film components at higher temperature, which resulted in better surface morphology [8].

### 3.5. Fourier Transform Infrared Spectroscopy

**Figure 5:**
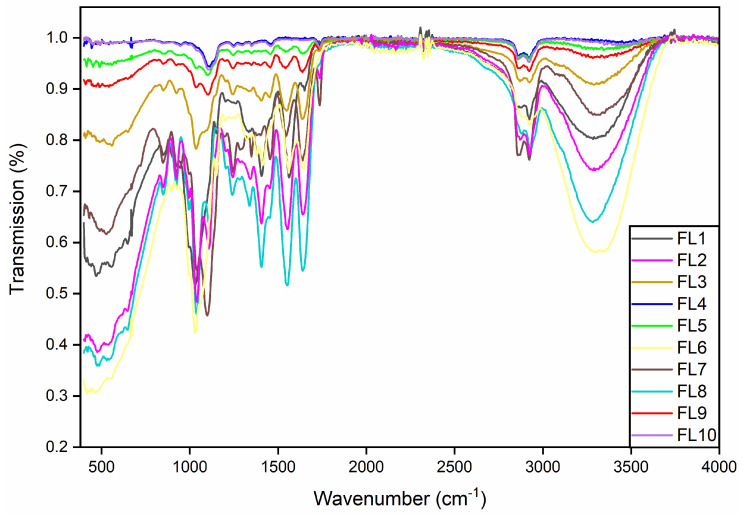
FTIR spectra of the EFs (FL1−FL10). FL1−FL5; EFs dried at 25 °C, FL6−FL10; EFs dried at 45 °C.

FTIR was performed to study the impact of GE and drying temperature on the chemical properties of the films. The transmission intensity and FTIR band patterns of all the samples are shown in Figure 5. FTIR analysis revealed chemical interactions between the various components of the films, i.e., CS, GE and GEO. Overall, the films showed similar characteristic peaks in accordance with other CS-GE based films studied before [34,35].

The peaks at 3292.3 cm^−1^ in the EFs are owing to the O-H stretching vibration, which joined together with N-H stretching [34,36]. This peak is flattened in samples (FL4, FL9, FL5 and FL10) and is more intense in the remaining samples. The shift in band intensity from a lower to higher number could be attributed to the decrease in bond length [37]. Peaks observed at the 2923.3 and 2879.5 cm^−1^ positions are attributed to the C-H stretching of the alkane groups of the CS or GE [34]. In addition, peaks observed at 1645 and 1562.1 cm^−1^ could be attributed to the carbonyl group stretching (C-O) and amino group (N-H) stretching vibration, respectively [36]. Moreover, the peaks observed at 1411 and 1051.4 cm^−1^ were ascribed to the N-H stretching [38] and C=O stretching vibration, respectively [39]. The characteristic bands at 846, and 923 cm^−1^ could be attributed to C–O–C stretching of the polysaccharide chain [35,40].

The GEO and GE added CS films, as well as the blank films, indicated more intense peaks at the fingerprint positions. The change in peak intensities between the samples could be associated with the difference in drying temperatures [41]. Overall, the FTIR analysis revealed that the band patterns and peak intensities of the blank films (FL1 and FL6) and GEO added CS films (FL2 and FL7) were found to be comparatively similar. On the other hand, the band patterns and peak intensities of the GE-GEO loaded CS films (FL4, FL5, FL9, and FL10) showed similarity. These findings suggest that the different drying conditions had less effect on the chemical properties of the composite films. Furthermore, the addition of a higher quantity of GE may also be responsible for the shift in peak intensities.

### 3.6. Thickness of the EFs

In the present work, an increase in the thickness of the edible has been observed from the FL1 to FL5 and FL6 to FL10 films (Table 3), except FL3 and FL8. It was also observed that composite films without GEO showed less thickness than those with GEO. This could be associated with a rise in solids content or because of the presence of peptide chains in gelatin, which ultimately restricted the formation of a compact film in the presence of the EO [42]. Additionally, it was found that the thickness of the films was decreased by increasing the drying temperature. This could be associated with an increase in the evaporation of water molecules and EO at 45 °C.

### 3.7. Mechanical Properties

Table 3 presents the mechanical characteristics of the films. Results obtained from mechanical testing suggested an increase in EAB values from FL1–FL5 and FL6–FL10 and higher TS values were observed for the CS-GE films without oil (FL3 and FL8). A higher level of amino acid can provide more stiffness and rigidity to gelatin-based films [43]. Furthermore, films dried at 45 °C showed relatively higher values of EAB and TS. Additionally, GEO loaded CS film (FL2 and FL7) samples showed comparatively lower TS values (*p* < 0.05) than the other films. This could be due to the effect of GEO in reducing the crystallinity of the samples. The addition of GEO can increase the amorphousness of the samples. The higher TS in CS-GE based films could be because of the higher amino acid content present in GE, hydrogen bonding between polyphenol and protein, and good compatibility among the film components, which resulted in film strengthening [44]. The increase of crystallinity in samples dried at 45 °C temperature could be due to the increase in the ordered arrangement of polymeric molecules at higher drying temperature conditions [33]. A higher concentration of amino acid can lead to formation of a more rigid as well as stiff gelatin film, as the triple helix structure of gelatin molecules is composed of the repetitive amino acid sequence: glycine–proline–hydroxyproline [45]. In addition, the GEO samples showed relatively lower TS values compared to the blank films, possibly because of the interference caused by their chemical components of GEO, with a polymer-protein (CS-GE) interaction in the film network resulting in discontinuity of the polymeric matrix, and a reduction in cohesive forces, as well as the strength of the films [45]. Thus, in the present work, TS increased slightly with the increase in GE (1–1.5%). The TS values of films dried at 45 °C were found to be greater than the films dried at 25 °C. This drop in TS at 25 °C could be due to the comparatively lower dissolution of oil in the film matrix [43]. The higher TS values at 45 °C could be due to the highly ordered hydrogen-bonded network structure [46].

Higher EAB values were observed in the CS-GE samples compared to films containing only CS (FL1, FL2, FL6 and FL7) due to the viscoelasticity provided by the gelling properties of GE. Moreover, the higher EAB value noted in GEO loaded CS-GE composite film was due to the ability of GEO to decrease the intermolecular interactions among gelatin polymer chains, and thus, encouraged film flexibility and chain mobility [47]. This could also be due to hydrogen bonding between the phenolic components of GEO and amino acids [48], which provided more flexibility to the films. Moreover, because of the plasticization effect of GEO, the GEO loaded samples showed greater EAB values [49].

Films dried at 45 °C showed lower Young modulus (Y_m_) values than those dried at 25 °C; this could be due to the increase in overall crystallinity, and the evaporation of a certain level of water molecules and essential oil at higher temperature. The decrease in Ym values from F1-F5 and F6-F10 could be due to the addition of gelatin and GEO. The addition of GEO may have reduced the overall crystallinity offered by the blank CS samples.

### 3.8. Water Vapor Permeability (WVP)

In comparison to gas molecules, water vapor possesses high permeability, thus it is a matter of concern for polymeric materials meant for food packaging, as a high transmission rate of water vapor can impact the food’s quality and its shelf life. The WVP for natural and biodegradable polymeric materials must be low to offer maximum resistance to water vapors and to also stop excessive water loss from the packed food into the environment [50]. However, the majority of natural and biodegradable polymeric materials exhibit poor water vapor barrier properties. Generally, many factors, such as surface morphology (e.g., defects, such as porosity and cracks), thickness, density, composition, number of layers, type of polymer and atmospheric temperature, as well as the relative humidity, determine the WVP of a polymeric material [51]. Additionally, the infiltration of small molecules across a polymeric barrier is determined by its diffusion rate and its solubility. Measuring the extent of vapor transmitted through a material can offer better understanding when assessing its suitability as a water-resistant coating material. Several barrier fillers or modifiers have been reported to improve the WVP [52,53]. As reported previously, the higher the GEO level in a polymeric film, the lower the WVP [54].

Current findings suggest that oil loaded films show lower WVP values than those without GEO. A blend of CS and GE showed a drop in the WVP values by forming a tortuous path across the film, improving the cohesiveness of the polymer via filling the empty spaces among the polymer chains and decreasing free space for water vapor transmission [55]. In addition, gelatin contains higher amounts of proline and arginine than bovine gelatin, which imparts more hydrophobicity to the polymeric network, and thus, decreases the permeability of water vapor across the films [43]. Drying temperature significantly affects the WVP of the material. In a groupwise comparison of samples, it was reported that WVP values decreased significantly with an increase in drying temperature except for the blank samples [56]. Our findings are in agreement with these previous studies, confirming that films dried at higher temperature show lower WVP values than those dried at lower temperature. This might be due to the change in overall crystallinity of polymers at higher temperature. Thus, a change in the drying conditions may influence the polymer architecture and arrangement resulting in changes in density or compactness of the polymer. On the other hand, an increase in the amount of gelatin has not shown a significant difference in WVP values. This could be due to the greater availability of free volume in the polymer matrix at higher concentrations, which may slightly restrict the transmission of water molecules across the films [57]. In the current work, the composite films (CS-GE) showed lower WVP values than the CS blank, because CS might have increased the crosslinking of gelatin [57]. Gelatin, due to the presence of high levels of hydrophilic amino acids, has shown greater WVP values [58]; however, in the presence of CS, GE exhibited low WVP. This behavior could be due to the increase in the crosslinking of gelatin.

### 3.9. Water Solubility

The water solubility of the films represents their ability to resist against water as well as their integrity in the presence of water [38]. CS is a weak base and is insoluble in water; however, gelatin-based films demonstrate low water resistance because of their hydrophilic nature [58]. The solubility of CS is dependent on pH; the solubility is due to the protonation of amino groups at pH 6 or less to produce cationic amine groups [59]. The water solubility of the samples is demonstrated in Table 3. It was found that the WS values were higher for the films dried at high temperature than those that were dried at the lower temperature, except FL9 and FL10. This is due to the evaporation of oil at high temperature conditions. The GEO loaded samples showed less water solubility than the CS blank because of its hydrophobic nature; and the evaporation of GEO will decrease the hydrophobicity of the samples. It was observed that the variation in the concentration of gelatin from 1 to 1.5% (*w*/*v*) did not impact the WS values significantly (*p* > 0.05). This could be due to the low bloom value of gelatin, which does not impact water solubility after increasing the concentration by 0.5% [60].

### 3.10. Moisture Content

Detailed information of the moisture content (MC) of the prepared films is given in Table 3. The MC values ranged from 40.21–30.47% for the films dried at room temperature and 30.22–23.22% for the films dried at a higher temperature. The GEO loaded samples showed less moisture content because the hydrophobic nature of oil prevents water absorption, and further, the free interstitial sites could be occupied with oil droplets. The composite films without GEO showed a higher moisture content than the GEO loaded CS films because of the presence of GEO. Similarly, to WS, the variation in the concentration of gelatin from 1 to 1.5% (*w*/*v*) did not impact the MC values significantly (*p* > 0.05).

### 3.11. Swelling Factor

The swelling degree of EFs dried at 25 °C varied from 89.21–177.11%, while the swelling degree of EFs dried at 45 °C varied between 67.16–128.16%, respectively. Gelatin containing films loaded with GEO showed greater SD values than the other samples (FL3 and FL8). This showed that the swelling behavior is greatly affected by the GE and GEO contents (Table 3). Elevation of swelling degree due to the addition of oil could be associated with the alteration of the microstructure during drying [61]. The change in the gelatin ratio increased the swelling degree of the films from FL4 to FL5 at both drying conditions. This finding is inconsistent with previous findings where changes in the gelatin ratio did not have much effect on the swelling behavior, possibly due to the change in the behavior of gelatin in the presence of GEO [62].

### 3.12. Transparency

Film transmittance is usually influenced by changes in the composition, compactness, type, grade, and arrangement of the polymers. Arrangement of the polymers impact the refraction index, and thus, limit the transmission of light across the matrix. As per the current finding, films dried at 45 °C showed more transparency than the films dried at 25 °C. This might be due to the evaporation of GEO and water molecules. In addition, blank CS films (F1, F6) showed more transparency than the GEO loaded and GE blended films. Addition of GE reduced the light transmittance; however, the incorporation of GEO had a more significant effect on light transmission. A decrease in transparency due to the addition of oil has been previously reported in several studies [63,64]. This could be due to the absorption of light by the chemical constituents of the oil [65]. Obstruction in light transmittance caused by GE could be because the amino acids (phenylalanine, tyrosine, and glutamine) present in porcine GE have a double bond structure. These amino acids contain more double bonds, which account for the absorbance of light, and thus, show lower light transmission values [66]. These findings indicate that GE in combination with essential oils demonstrate better UV-reflecting properties due to the aromatic amino acids, which are capable of absorbing UV light.

### 3.13. Total Phenol Content

**Figure 6:**
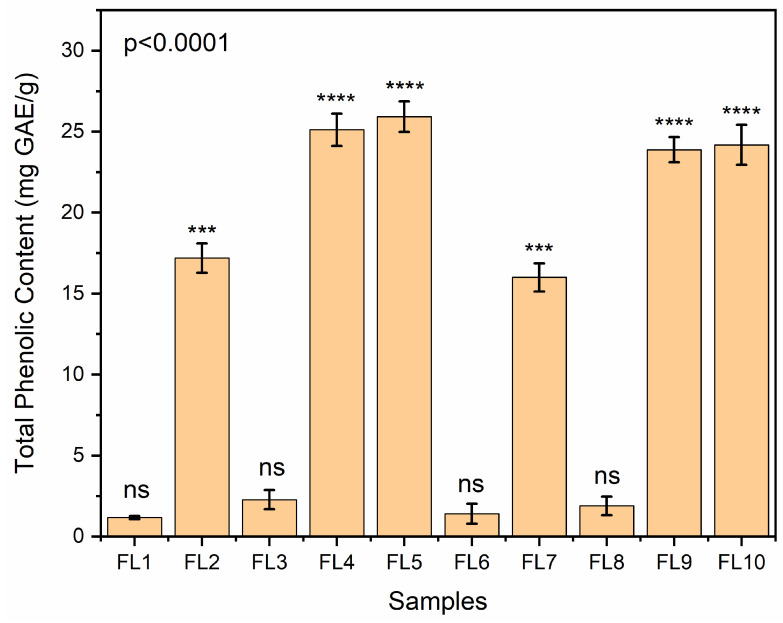
Total phenolic content analysis of edible films (FL1–FL10), dried at various temperature. Samples FL1–FL5 were dried at 25 °C and samples FL6–FL10 were dried at 45 °C. Asterisks on bars reveal significant variations between the groups; ns = not significant, *** *p* < 0.0002, **** *p* < 0.0001.

The total phenolic content of samples is demonstrated in Figure 6. CS blank films revealed a TPC of 1.75 mg GAE/g film. This finding corroborates with the findings described by Ruiz-Navajas et al. 2013. This could be due to the chromogen generation (blue color complex formation) with non-phenolic reducing substances [25,67]. GEO loaded CS-GE samples showed more phenolic content than blank CS and CS-GE samples. EFs samples without GEO (FL1, FL3, FL6 and FL8) exhibited neglected amount of phenol content and thus were labelled as ns; non-significant (Figure 6). Overall, the TPC content of GEO loaded films dried at the low temperature (FL2, FL4 and FL5) was found to be higher than the films dried at the high temperature. However, differences in the TPC content of the films dried at both conditions was found to be lower and insignificant. This phenomenon was also previously observed by Monica A. M et al. 2009 [68] who stated that the phenolic content converts into other chemical motives, such as chlorogenic and neochlorogenic acid, at a higher drying temperature. The small difference in the TPC content could possibly be due to changes in the structural configuration of the polymer or more crystallinity of the films, which could have made them denser and more compact with fewer pores, resulting in the entrapment of more essential oil droplets into the matrix. No significant difference was found between the TPC content of CS (FL1, FL6) and CS-GE (FL3, FL8) without GEO due to the presence of CS in all samples.

### 3.14. Antioxidant Assay

**Figure 7:**
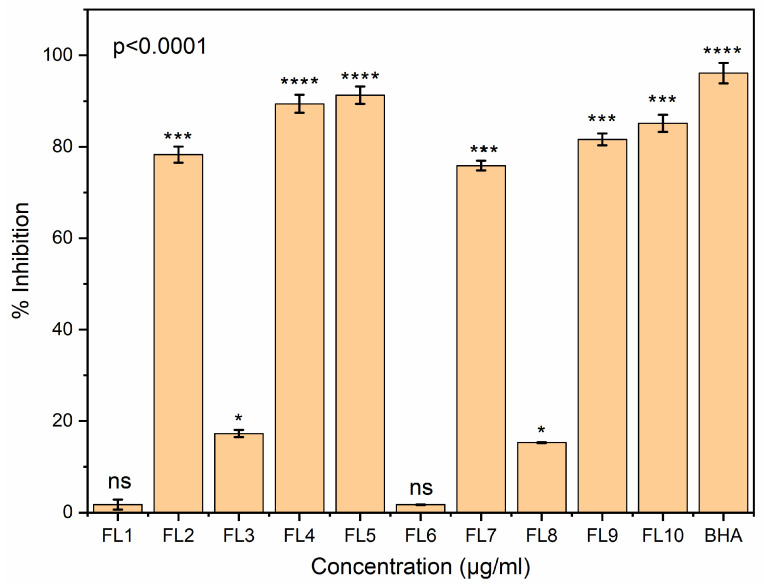
The antioxidant potency of FL1–FL10 was assessed in comparison with standard BHA, using the DPPH method. Bars correspond to the mean values ± standard variations. Asterisks on bars reveal significant variations between the groups; ns = not significant, * *p* < 0.0332, *** *p* < 0.0002, **** *p* < 0.0001.

**Figure 8:**
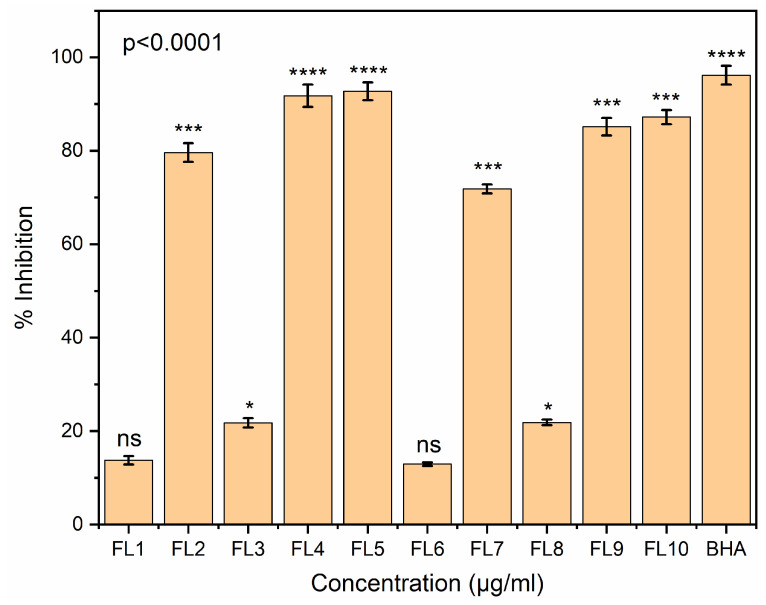
Antioxidant potency of FL1–FL10 evaluated in comparison with standard BHA determined by the ABTS method. Bars correspond to the mean values ± standard variations. Asterisks on bars reveal significant variations between the groups; ns = not significant, * *p* < 0.0332, *** *p* < 0.0002, **** *p* < 0.0001.

Antioxidant property assessment is one of the most important parameters used to assess the capability of active packaging material to prevent food oxidation due to intrinsic and extrinsic factors. In the present study, the antioxidant activities of CS, CS-GE and CS-GE-GEO samples (dissolved in 1% acetic acid and then added to 15 mL methanol solution (10%)) were assessed by DPPH and ABTS assays. FL1 and FL6 samples contain no oil due to which these samples exhibited non-significant (ns) antioxidant potential. As demonstrated in Figure 7 and Figure 8, films dried at the low temperature showed more antioxidant activity than those dried at the high temperature. This could be associated with the conversion of some of the phenolic compounds to other derivatives at high temperature condition. Furthermore, the antioxidant effect of GEO could be due to the presence of phenolic components, such as eugenol, shogaols, zingerone, gingerdiols, and gingerols [69].

The reduced antioxidant activity at high temperature could also be due to changes in the structural configuration of the polymer, due to which, the film became more dense and less porous, resulting in the entrapment of GEO, and thus, limiting the release of GEO. In both assays, the CS-GE films showed more antioxidant activity than the CS blank films. This could be attributed to the hydrolysis of GE, resulting in the release of antioxidative peptides rich in hydroxyproline, and proline. Additionally, a change in the antioxidant properties of these samples could be ascribed to the change in the solubility of the CS and CS-GE films at two different drying conditions [70].

GEO loaded films showed more antioxidant effects than blank CS and CS-GE samples because of the presence of the phenolic components in the essential oil loaded in these films. Furthermore, there was no prominent difference found between the antioxidant activity of the CS-GE-GEO films with 1 and 1.5% GE. It was observed that FL4 and FL5 demonstrated almost comparable antioxidant effects to BHA, which could be due to the synergistic interactions between the phenolic components of GEO and CS.

## 4. Conclusions

The fabrication of biopolymer based EFs for food packaging holds several advantages over synthetic packaging materials. The process parameters, i.e., drying temperature, as well as the composition of the films (such as the type and concentration of the polymers), significantly impact the chemical, physical, mechanical, and antioxidant properties of the films. Blending chitosan with other polymer and hydrophobic core materials, such as essential oil, significantly enhances its characteristics, and eventually, its applications in food packaging. In the current study, we fabricated GEO loaded, chitosan based EFs with different concentrations of gelatin (1–1.5%) at different drying conditions (25 °C and 45 °C), and studied their physical, chemical, mechanical and antioxidant properties. We concluded that chitosan-gelatin based composite edible films incorporated with ginger EO demonstrated better physio-chemical properties at a higher drying temperature and good antioxidant potential was observed at a lower drying temperature. An increase in gelatin concentration from 1 to 1.5% did not show any significant impact on its physical, chemical, and antioxidant properties. The application of these oil loaded composite films can be utilized in food packaging as they exhibit better optical, barrier, mechanical, and antioxidant properties. Furthermore, the material fabricated in this current study requires further attention in terms of the assessment of its antimicrobial properties, its performance in food applications, and its sensory attributes.

## Figures and Tables

**Table 1:** Composition of the blank and composite CS-GE films (FL1–FL10).

Sample Codes	Films Composition	Drying Temperature
FL1	CS (1%)	25 °C
FL2	CS (1%) + GEO (1.5%)	
FL3	CS (1%) + GE (1%)	
FL4	CS (1%) + GE (1%) + GEO (1.5%)	
FL5	CS (1%) + GE (1.5%) + GEO (1.5%)	
FL6	CS (1%)	45 °C
FL7	CS (1%) + GEO (1.5%)	
FL8	CS (1%) + GE (1%)	
FL9	CS (1%) + GE (1%) + GEO (1.5%)	
FL10	CS (1%) + GE (1.5%) + GEO (1.5%)	

**Table 2:** Visual assessment of the chitosan and gelatin-based films with and without EO.

Sample Codes	Components	Drying Temperature	Visual Attributes
FL1	CS	Films dried at 25 °C	Less transparent than FL6, inflexible, fragile, non-sticky, difficult to peel, brittle, stiff
FL2	CS-GEO		Same characteristics as FL1 except slight yellowness in appearance
FL3	CS-GE		Less transparent than FL8 and FL1, sticky, shrinkage, difficult to peel and handle
FL4	CS-GE(1%)-GEO		Showed same characteristics as FL3
FL5	CS-GE(1.5%)-GEO		Poor transparency, cracks, pores, brittle, fragile and difficult to peel out, slightly adhesive
FL6	CS	Films dried at 45 °C	More transparent and flexible than FL1, less fragile, brittle, and stiff, easy to peel and non-sticky
FL7	CS-GEO		Same characteristics as FL6 except slight yellowness in appearance
FL8	CS-GE		More transparent than FL3, however, transparency was less than FL6 and less sticky, brittle and fragile, more flexible than FL3, easy to peel and handle
FL9	CS-GE(1%)-GEO		More transparent than FL4, non-brittle, non-fragile, flexible, easy to peel, less sticky, showed no shrinkage from edge
FL10	CS-GE(1.5%)-GEO		More transparent than FL5, no cracks, pores, easy to peel out, non-sticky

**Table 3:** Mechanical properties of WVP, thickness, EAB, TS, Ym, SD, WS, MC, and transparency of the samples.

Formulations	Thickness (μm)	TS (MPa)	EAB (%)	Ym (MPa)	WVP (×10^−12^ g·cm/cm^2^·s·Pa)	WS (%)	MC (%)	SD (%)	Transparency (%)
FL1	56.12 ± 1.1 ^b^	42.11 ± 1.33 ^c^	21.22 ± 5.12 ^f^	82.21 ± 5.11 ^a^	4.73 ±0.05 ^a^	38.26± 5.17 ^b^	40.21 ± 3.05 ^a^	89.21 ± 6.03 ^d^	43.21 ± 3.17 ^c^
FL2	58.11 ± 2.1 ^b^	40.77 ± 2.41 ^c^	28.34 ± 3.17 ^e^	73.11 ± 3.10 ^b^	3.56 ± 0.02 ^b^	26.24 ± 5.22 ^d^	35.11 ± 2.11 ^b^	121.17 ± 4.05 ^c^	17.11 ± 1.01 ^f^
FL3	55.22 ± 1.5 ^b^	44.14 ± 2.32 ^c^	29.11 ± 1.20 ^e^	67.55 ± 4.16 ^c^	3.82 ± 0.03 ^b^	31.26± 3.15 ^c^	41.55 ± 3.08 ^a^	117.37 ± 7.13 ^c^	26.55 ± 2.02 ^d^
FL4	65.11 ± 1.2 ^a^	42.11 ± 1.23 ^c^	47.11 ± 2.10 ^c^	35.89 ± 6.10 ^e^	3.14 ± 0.01 ^c^	26.58 ± 2.10 ^d^	28.89. ± 1.01 ^c^	151.32 ± 2.55 ^b^	16.89. ± 1.01 ^f^
FL5	68.77 ± 1.6 ^a^	43.38 ± 1.88 ^c^	51.24 ± 4.11 ^c^	33.47 ± 2.20 ^e^	3.03 ± 0.04 ^c^	23.26± 1.19 ^d^	30.47 ± 1.22 ^c^	177.11 ± 8.11 ^a^	18.47 ± 1.07 ^f^
FL6	41.23 ± 2.4 ^d^	66.24 ± 3.17 ^a^	24.26 ± 3.17 ^e^	77.22 ± 6.22 ^b^	4.57 ± 0.04 ^a^	46.22 ± 5.21 ^a^	30.22 ± 3.18 ^c^	67.16 ± 2.01 ^g^	64.22 ± 2.21 ^a^
FL7	47.77 ± 1.4 ^d^	54.21 ± 1.34 ^b^	44.24 ± 2.10 ^d^	61.47 ± 3.24 ^c^	3.49 ± 0.02 ^b^	32.34 ± 8.23 ^c^	25.87 ± 2.14 ^d^	82.11 ± 7.18 ^e^	43.47 ± 4.17 ^c^
FL8	42.23 ± 1.3 ^d^	68.87 ± 4.01 ^a^	61.26 ± 3.01 ^b^	48.22 ± 4.12 ^d^	1.21 ± 0.02 ^d^	43.11 ± 6.11 ^a^	29.22 ± 1.18 ^c^	75.16 ± 4.08 ^f^	51.22 ± 2.05 ^b^
FL9	47.77 ± 2.1 ^c^	55.11 ± 5.36 ^b^	82.24 ± 6.1 ^a^	32.47 ± 5.11 ^e^	1.13 ± 0.02 ^d^	27.11 ± 3.24 ^d^	24.47 ± 2.10 ^d^	91.11 ± 3.01 ^d^	29.47 ± 1.12 ^d^
FL10	51.11 ± 1.5 ^c^	58.32 ± 3.48 ^b^	87.26 ± 3.12 ^a^	25.22 ± 1.11 ^f^	1.11 ± 0.05 ^d^	24.14 ± 2.12 ^d^	23.22 ± 1.05 ^d^	128.16 ± 5.22 ^c^	20.22 ± 1.05 ^e^

Results are shown as the mean value ± standard deviation. Superscripts with different letters signify significant differences (*p* < 0.05).

## Data Availability

Not applicable.
